# Graphical Approach to Model Reduction for Nonlinear Biochemical Networks

**DOI:** 10.1371/journal.pone.0023795

**Published:** 2011-08-25

**Authors:** David O. Holland, Nicholas C. Krainak, Jeffrey J. Saucerman

**Affiliations:** Department of Biomedical Engineering, Robert M. Berne Cardiovascular Research Center, University of Virginia, Charlottesville, Virginia, United States of America; Mount Sinai School of Medicine, United States of America

## Abstract

Model reduction is a central challenge to the development and analysis of multiscale physiology models. Advances in model reduction are needed not only for computational feasibility but also for obtaining conceptual insights from complex systems. Here, we introduce an intuitive graphical approach to model reduction based on phase plane analysis. Timescale separation is identified by the degree of hysteresis observed in phase-loops, which guides a “concentration-clamp” procedure for estimating explicit algebraic relationships between species equilibrating on fast timescales. The primary advantages of this approach over Jacobian-based timescale decomposition are that: 1) it incorporates nonlinear system dynamics, and 2) it can be easily visualized, even directly from experimental data. We tested this graphical model reduction approach using a 25-variable model of cardiac β_1_-adrenergic signaling, obtaining 6- and 4-variable reduced models that retain good predictive capabilities even in response to new perturbations. These 6 signaling species appear to be optimal “kinetic biomarkers” of the overall β_1_-adrenergic pathway. The 6-variable reduced model is well suited for integration into multiscale models of heart function, and more generally, this graphical model reduction approach is readily applicable to a variety of other complex biological systems.

## Introduction

Biological systems are inherently complex, with regulation and feedback at numerous spatial, temporal and functional scales. As a result, multiscale computational models are essential for understanding systems properties not attributable to any individual component [1]. Multiscale models such as those in the Physiome Project [2], [3] have been developed for many areas including the cardiovascular system, respiratory system, cancer and angiogenesis [4]. Recent models now also span from protein structure to cellular function [5], [6]. One of the most formidable challenges now facing multiscale modeling efforts is model reduction [7]. Model reduction will be crucial for computational feasibility [8], but may also play important roles in easing model implementation, reducing the number of free parameters [1], and extracting conceptual insights from complex systems.

Most model reduction approaches use a form of timescale decomposition, which has its foundation in singular perturbation theory [9]. Timescale decomposition is used in a wide range of fields including chemical kinetics [10], [11], [12] , flight guidance [13], structural dynamics [14], and weather forecasting [15]. If fast species are well separated from slow species, fast timescale species can be assumed to be at quasi-steady state and replaced with algebraic equations, while the slow species are retained in the reduced model [12], [16]. However, this approach raises a challenge: how does one determine whether there is sufficient timescale separation, and which species are “fast” or “slow”? In most cases these decisions require significant *a priori* knowledge, restricting the use of timescale decomposition to compact and well-studied systems [12].

To address this challenge, a number of systematic timescale decomposition approaches have been developed that involve linearizing the system and performing decompositions of the Jacobian matrix [9], [11], [12], [16], [17], [18], [19]. Jacobian analysis is scalable, can be performed quickly, and provides the distribution of timescales and the species that participate at each timescale [19]. However, Jacobian-based approaches also have limitations: they most often analyze a linearized steady-state rather than overall nonlinear dynamics; they involve complex matrix decompositions in which biological relevance may be obscured; and a given timescale may involve many different species that are not functionally related. A second challenge to model reduction is raised after timescale decomposition is performed. The reduced model is a differential-algebraic system, where algebraic equations are implicit and may have multiple roots, complicating numerical solution [7], [9].

Here, we introduce a graphical approach to timescale decomposition based on phase-plane hysteresis. This approach allows for intuitive yet systematic identification of timescale separation, accounting for nonlinear dynamics of the system. We pair this analysis with a “concentration-clamp” approach for estimating explicit steady-state relationships among rapidly equilibrating species, avoiding the numerical difficulties of implicit algebraic equations. We tested this graphical model reduction approach using a 25-variable differential-algebraic model of cardiac β_1_-adrenergic signaling [20], [21]. This signaling network plays a central role in cardiac regulation and disease [22], but the complexity of the original model limits its inclusion into multiscale models of the heart. The model reduction approach was used to obtain a 6-variable reduced β_1_-adrenergic model that retains good predictive abilities even to new perturbations not used in the model reduction. Thus these 6 signaling species are “kinetic biomarkers” whose measurement captures the overall dynamics of the overall β_1_-adrenergic pathway. In addition, we expect that the graphical model reduction approach will be readily applicable to a variety of other complex biological systems.

## Results

### A Toy Model Example

**Figure 1:**
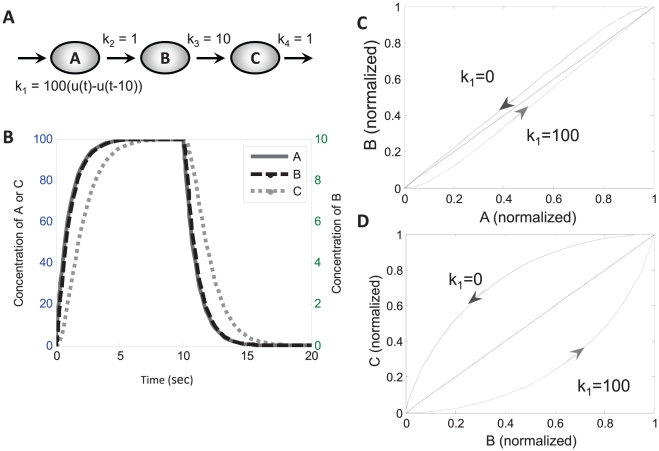
Phase portraits reveal timescale separation in a toy model. (A) Schematic of toy model, showing both the species and the reaction constants. Input parameter “k_1_” is set to 100 µM^−1^s^−1^ between 0–10 seconds. (B) Predicted time-courses of the toy model. The relationship between A and B is “fast”; they equilibrate quickly so that the concentration of B is always close to one-tenth of A. The relationship between B and C is slow, evidenced by the time lag between the two time-courses. (C) Normalized phase plane of B vs. A. Here the area between the phase plots is small as their trajectories stay close to their steady-state relationship (the dotted line). (D) Normalized phase plane of C vs. B. The phase trajectories exhibit greater hysteresis, creating a large area in between plots.

To illustrate the basic principles of graphical timescale separation, we built a toy reaction model consisting of a linear irreversible pathway with 3 species (A, B and C) and 4 reactions (see Figure 1A). The model was implemented as 3 ordinary differential equations using first-order mass action kinetics:
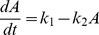
(1)

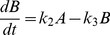
(2)

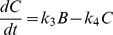
(3)where k_1_ = 100(u(t)-u(t-10)) µM^−1^ s^−1^, k_2_ = 1 s^−1^, k_3_ = 10 s^−1^, k_4_ = 1 s^−1^, and u(t) is the unit step function. As shown in Figure 1B, setting k_1_ = 100 stimulates coincident increases in A and B, while C increases more slowly. These species decay with a similar kinetic pattern when t>10 sec, when k_1_ is returned to 0 µM^−1^s^−1^.

Phase portraits for each pair of variables were computed, normalizing each variable by x_norm_(t) = (x(t)-x_min_)/(x_max_-x_min_). The corresponding normalized phase portrait for A vs. B (Figure 1C) encloses a fairly small area, with little hysteresis. The steady-state A vs. B relationship (dashed line) can be determined by a concentration clamp procedure (see Materials and Methods) where A is fixed and steady-state B is determined. Because the A vs. B phase loop is well approximated by the steady-state concentration clamp, the A vs. B relationship can be considered to be “fast”. In contrast, the phase portrait for B vs. C (Figure 1D) exhibits a larger area (greater hysteresis) and is therefore considered “slow”. We will attempt to discriminate between “fast” and “slow” relationships to guide model reduction. To simplify this toy model, the “fast” dynamics between A and B can be replaced by their steady-state relationship without significantly affecting the overall system dynamics. Thus Equation 2 reduces to B = (k_2_/k_3_)A.

### Timescale Separation in the β_1_ Adrenergic Signaling Network

**Figure 2:**
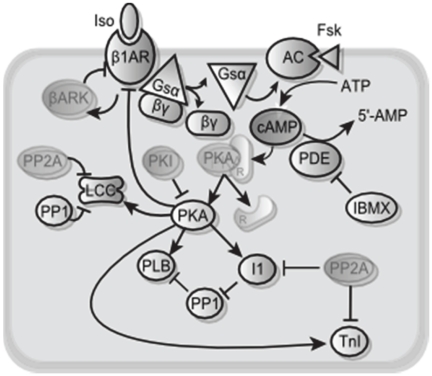
Schematic of 25-Variable β_1_-adrenergic signaling model. The opaque species in this schematic are state variables requiring either ordinary differential equations or implicit algebraic equations. The faded species are calculated by explicit algebraic equations based on state variables.

To evaluate this graphical timescale separation approach for a more realistic system, we examined a well-validated model of the cardiac β_1_ adrenergic signaling network [20], [21]. The β_1_ adrenergic signaling network regulates key aspects of heart rate and contractility [22], primarily via cyclic AMP (cAMP), protein kinase A (PKA) and PKA substrates such as phospholamban (PLB) and troponin I (TnI). This model is a differential-algebraic system of equations with 25 state variables (12 ODEs and 13 implicit algebraic equations), shown schematically in Figure 2. Parameters and initial conditions are provided in Table S1 and Table S2. Full equations are provided in the supplement to reference [21]. Model code is available in MATLAB and CellML formats (Dataset S1). The CellML code was generated using Cellular Open Resource [23] by modifying the version from the CellML repository [24], [25]. While this model is only of moderate size, the number of parameters and difficulty solving implicit algebraic equations indicates that a reduced model would be useful for embedding in large-scale models of heart physiology. In addition, model reduction may make nonlinear dynamic analyses more tractable, potentially leading to additional systems insights. The aim of model reduction was twofold: to eliminate the fast ODEs and make the implicit algebraic equations explicit.

**Figure 3:**
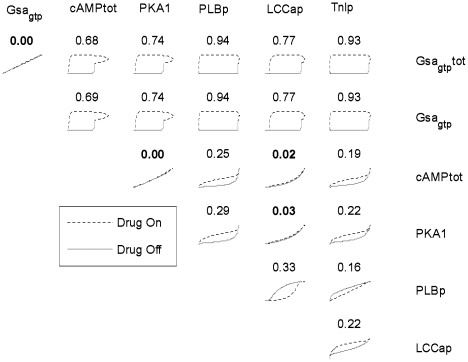
Global phase portrait of the β_1_-adrenergic network. While the global phase portrait is a 25-variable dimension space, 2D slices can be taken that illustrate timescale separation between pairs of state variables. Here, 21 illustrative normalized phase portraits are shown, with all 210 portraits shown in Figure S1. Above each portrait, a normalized area of the enclosed loop is calculated that quantifies the degree of timescale separation. The phase portrait area can range from 0 to 1, with 0 indicating no timescale separation.

Similar to the toy model, the β-adrenergic model was stimulated by a transient input, in this case isoproterenol (Iso). The time-course for each variable was normalized from zero to one using x_norm_(t) = (x(t)-x_min_)/(x_max_-x_min_), and a global phase portrait was created of all pairwise combinations of state variables. Normalizing timecourses allows quantitative comparisons between individual phase portraits, independent of the magnitude of a particular signal. Figure 3 shows 21 representative normalized phase portraits, while Figure S1 shows all 210 phase portraits. For each individual phase portrait, the encompassed area (hysteresis) was computed (see Methods) and displayed above each individual portrait. Again, large phase portrait areas correspond to greater timescale separation between the two variables, and smaller areas correspond to less timescale separation.

**Figure 4:**
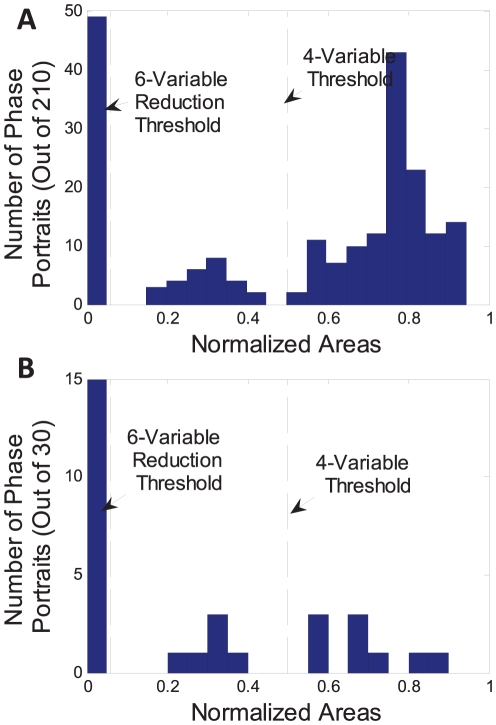
Histogram of phase portrait areas reveals timescale separation. (A) Histogram of phase portrait areas for all relevant species against each other (210 portraits). The histogram reveals 3 distinct modes representing fast (area <0.05), medium (0.05< area <0.5) and slow (area >0.5) relationships. (B) The 3 distinct timescales are still apparent when examining only adjacent relationships, in which one species is used directly to calculate another. These 30 phase portraits are the most biologically relevant. The fast relationships from this second histogram were reduced to obtain a simplified 6-variable model. Both fast and medium relationships were reduced when creating a further simplified 4-variable model.

As shown in Figure 4A, a histogram of phase portrait areas reveals a trimodal distribution of timescale separation in the network. The “fast” mode contains areas of 0.05 or less. The medium-speed mode has areas between 0.05 and 0.5, and the slow-speed mode contains areas greater than 0.5. But these portraits contain many indirect relationships that, while physiologically relevant, are not needed for model reduction (e.g. A vs. C in the toy model above). Figure 4B shows a histogram including only the 30 direct relationships where one species concentration was used to directly calculate the other. The trimodal distribution of timescales is still evident in the direct relationships.

### Reduced-order models of β_1_-adrenergic signaling

**Figure 5:**
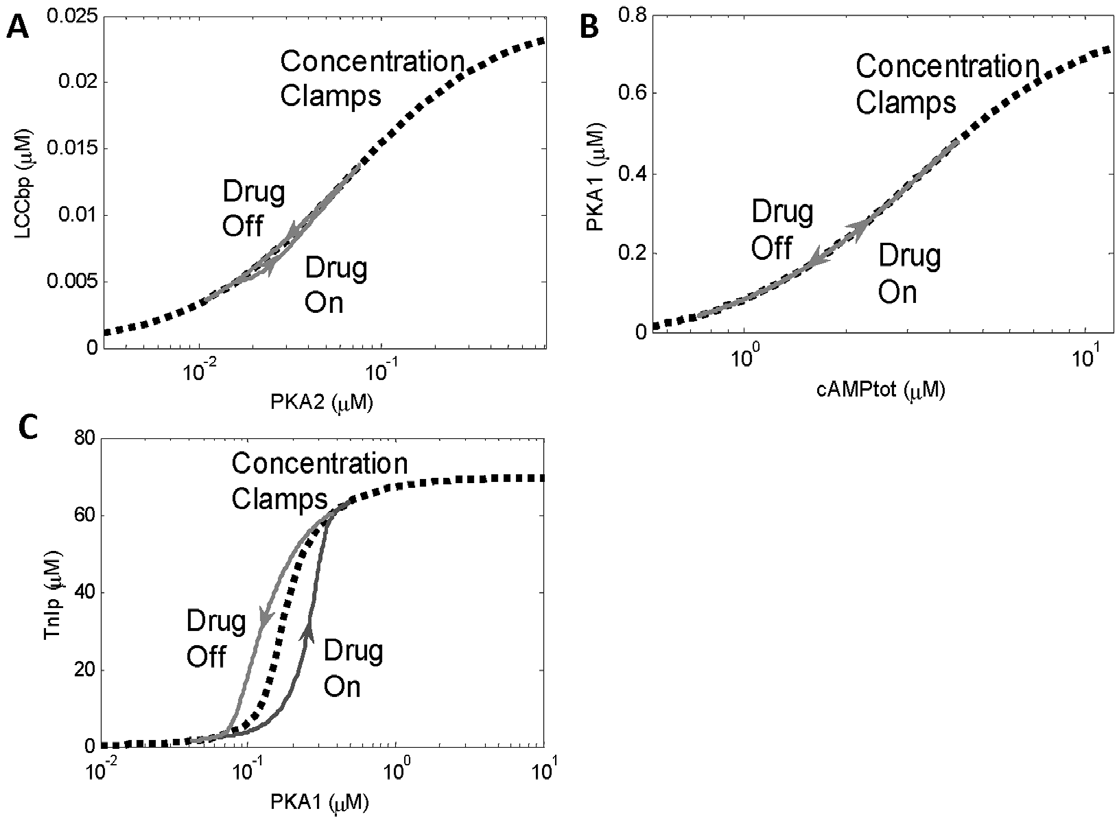
Concentration clamps used to approximate steady-state relationships. One species was held constant for while the steady-state value of the other was recorded. This was repeated for a range of concentrations. (A) Phase portrait for a fast relationship between PKA2 and LCCbp, used to create the 6-variable model. The area between phase plots is relatively small and thus can be reduced algebraicly by performing a steady-state concentration clamp. (B) Phase portrait for a medium relationship between PKAI and TnIp, which was retained in the 6-variable model but reduced in the 4-variable model. There is more disparity between the concentration clamp curve and the phase plots, creating more error. (C) Phase portrait for an implicit algebraic relationship between cAMPtot and PKAI. Concentration clamps were used to determine an explicit relationship between these variables.

Because Figure 4 shows clear separation between the timescales, this provides a guideline for which relationships in the system can be reduced. Figure 5 shows example phase portraits and their corresponding steady-state relationships determined by a computational procedure termed a “concentration clamp” during 1 µM Iso (see Materials and Methods). PKA2 vs. LCCbp (Figure 5A) and cAMPtot vs. PKA1 (Figure 5B) both have small phase portrait areas and are well-approximated by their steady-state relationships. In contrast, PKA1 vs. TnIp (Figure 5C) has an intermediate area (“medium” timescale separation), suggesting that reduction of this relationship may not be appropriate.

**Figure 6:**
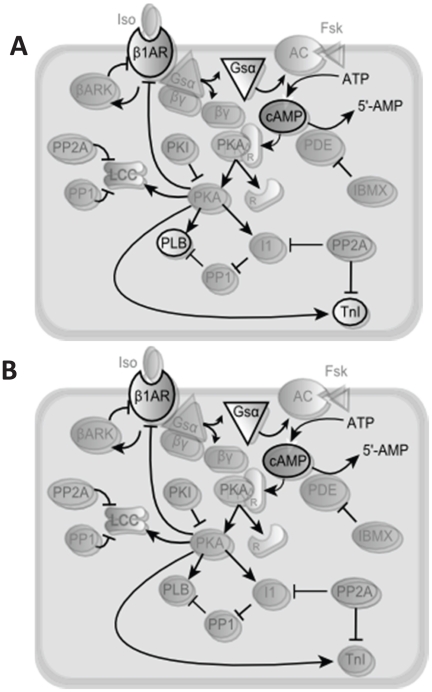
Schematics of A) 6-variable and B) 4-variable reduced models. The species calculated with differential equations are opaque, while the rest are faded. Compare with the 25-variable schematic (Figure 2). The 4-variable model has two less differential equations, represented by the further fading of TnI and PLB. Reduced (faded) species from the original 25-variable model are still predicted in the reduced models, but using explicit algebraic rather than differential or implicit algebraic equations.

Direct relationships in the fast mode (“low” timescale separation) were converted from differential equations or implicit algebraic equations to explicit algebraic relationships, reducing from 25 state variables to 6 (Figure 6A). For each of the reduced relationships, parameter estimation was used to fit an explicit algebraic equation to the corresponding concentration clamp. To evaluate the sensitivity of this approach to the choice of timescale separation threshold, we also chose a second threshold that reduces both “fast” and “medium” relationships. This second reduced model eliminates differential equations for TnIp and PLBp, resulting in 4 differential equations (Figure 6B). MATLAB code and CellML files for both 6-variable and 4-variable models are provided in Dataset S1. Parameters and initial conditions for 6-variable and 4-variable models are provided in the Table S3 and Table S4.

To test the prediction accuracy of the 6-variable and 4-variable reduced models, timecourses were computed under transient application of 1 µM Iso. Six variables were excluded from this analysis, either because they were fixed during this simulation protocol (forskolin, IBMX, phosphodiesterase) or they were removed in the reduced models (β1AR_free_, Gsα_gdp_, Gsβγ). Prediction error for each variable is shown in Table 1. For the 6-variable reduced model, the variable with the highest average error was PLBp with 3.8%, and the overall average error was 1.5%. The 4-variable model included the reduction of the calculation of PLBp and TnIp, increasing their mean errors to 11.5% and 24.5% respectively. The other concentrations did not significantly change between the 6-variable and 4-variable reduced models, but the 4-variable model's average error increased to 3.0%.

**Table 1:** Mean error of 6- and 4-variable reduced models compared with original model.

Species Name	Mean Error (%)	
	*6-Variable Model*	*4-Variable Model*
Ligand	0.4	0.4
Gs	0.1	0.1
β1ARtot	0.2	0.2
β1ARd	0.9	0.9
β1ARp	0.5	0.5
Gsαgtptot	0.3	0.3
Gsαgtp	1.7	1.7
AC	0.1	0.1
cAMPtot	1	1
cAMPfree	1.7	1.6
PKA1	2.6	2.6
PKA2	1.3	1.3
PLBp	3.8	**11.5**
Inhib1ptot	2.7	2.7
Inhib1p	3.1	3.1
PP1	0.2	0.2
LCCap	2.2	2.2
LCCbp	2	2
TnIp	3.6	**24.5**
**Mean**	**1.5**	**3**

Errors for 19 of the original 25 state variables are shown. Not shown are Forskolin and IBMX (set to 0 µM for most conditions), PDE, β1AR_free_, Gsα_gdp_, and Gsβγ (which were removed from or no longer necessary for the models).

**Figure 7:**
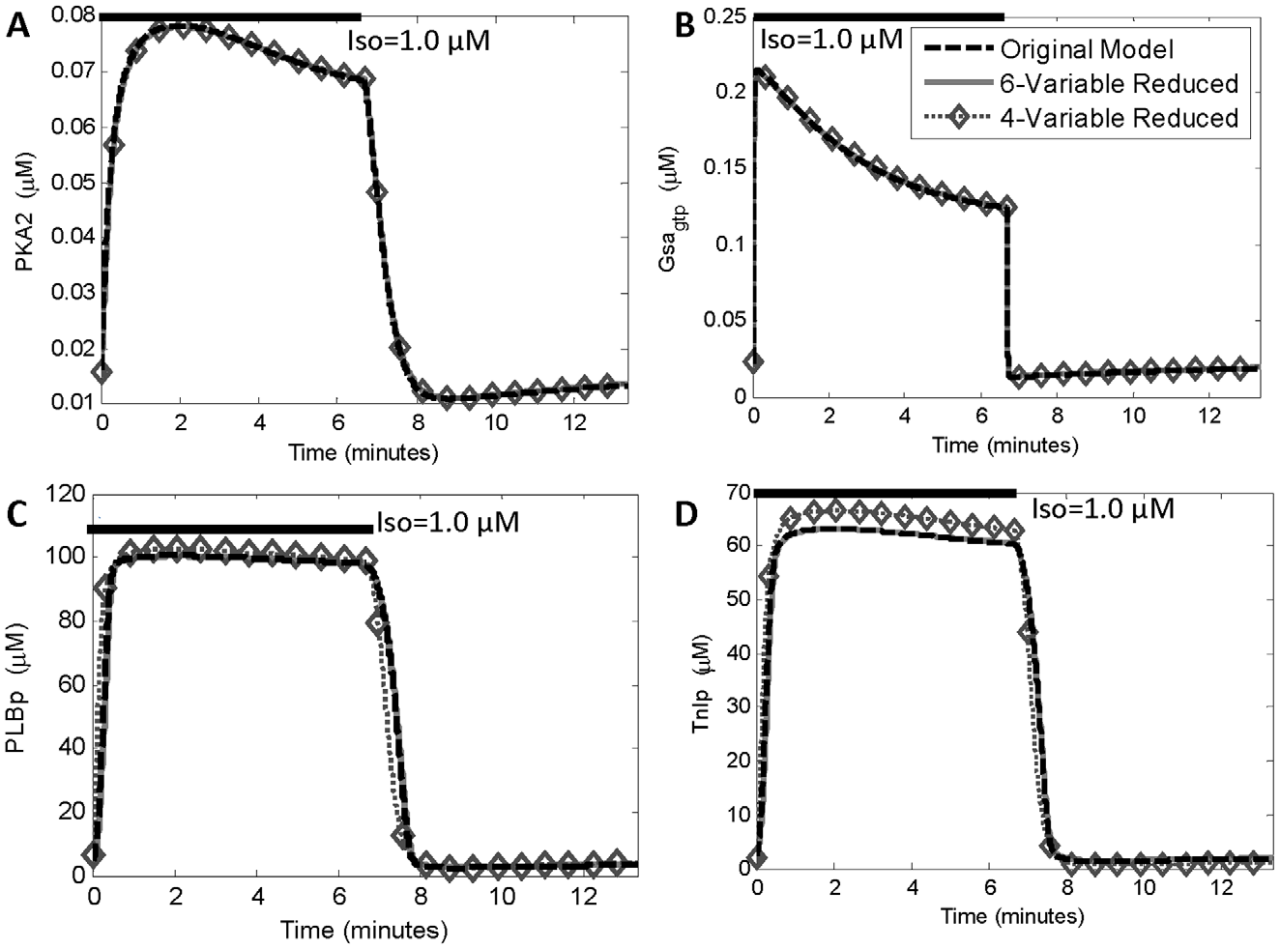
Accurate time-courses from 6-variable and 4-variable reduced models. (**A**), (**B**) Predictions of PKA2 and Gsa_gtp_ in response to transient 1 µM isoproterenol (0–7 minutes), plotted for original (25-variable) and reduced (6 or 4-variable) models. (**C**), (**D**) Predictions of PLBp and TnIp to transient isoproterenol exposure. Note that PLBp and TnIp were reduced in the 4-variable model. These reductions were less accurate, although the time-courses are still similar for all three models. Note that model reductions were performed with 1 µM ISO, so this is not an independent test of predictive capability.

Example timecourses for the original (25-variable) and reduced (6 and 4-variable) models are shown in Figure 7. For all timecourses, the 6-variable model was quite consistent with the dynamics from the original 25-variable model. Time-courses for the 4-variable model show that PLBp and TnIp were overestimated during drug application and underestimated during drug withdrawal, consistent with the deviation from steady-state observed in their respective phase portraits (e.g. Figure 7).

**Figure 8:**
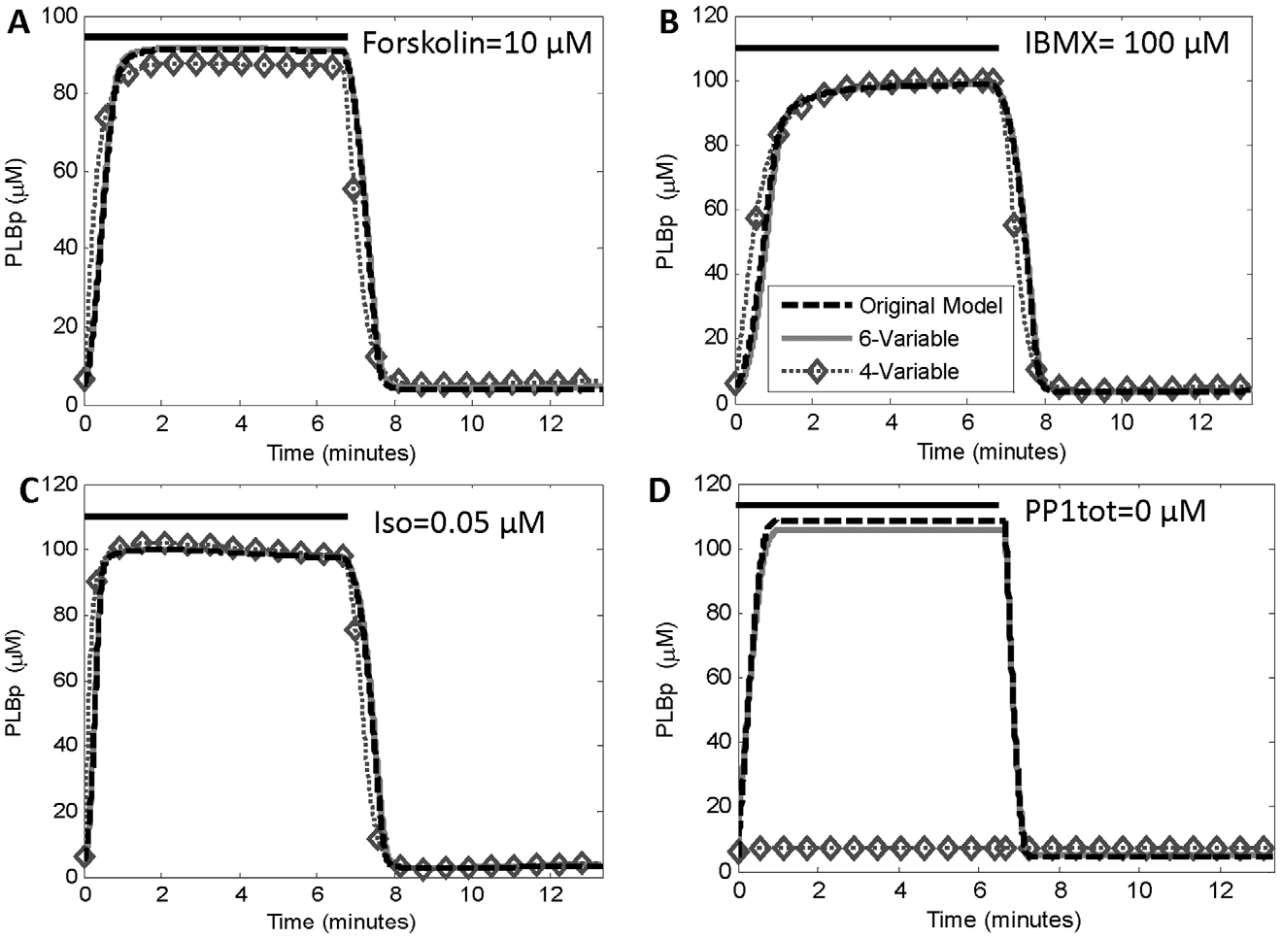
Predictive accuracy of 6-variable model retained during new prturbations. (**A**) Time-courses of phosphorylated phospholamban (PLBp) in response to transient exposure to 10 µM forskolin, which directly activates adenylate cyclase (AC). (**B**) Time-courses of each model with transient exposure to 100 µM IBMX, which directly inhibits PDE. (**C**) Time-course of each model with ISO = 0.05 µM instead of 1.0 µM. (**D**) Time-course of each model when PP1 is completely inhibited (PP1tot = 0) transiently. The 4-variable model did not respond to PP1 perturbation since the parameter representing total PP1 was eliminated during the reduction of PLBp.

To test the performance of the model under perturbations not used during model reduction, the models were run using four additional conditions. The first involved the transient application of 10 µM forskolin (absent from the previous runs) instead of β_1_AR ligand (Figure 8A). Similarly, the next perturbation was transient exposure of 100 µM IBMX, which inhibits phosphodiesterase (PDE) (Figure 8B). The third perturbation tested the model under the application of a smaller concentration of 0.05 µM Iso, as opposed to 1.0 µM (Figure 8C). The fourth perturbation involved inhibiting phosphatase-1 (Figure 8D). For the first three perturbations, the 6-variable model performed very well while the 4-variable model exhibited modest error, as expected. But during phosphatase inhibition, the 4-variable reduced model did not respond because phosphatase-1 was no longer used to predict PLBp in this model.

Most current timescale decomposition methods involve analysis of the Jacobian matrix at a particular model steady-state [19]. To compare our graphical phase-portrait approach to the Jacobian approach, we computed the 25x25 Jacobian of the original model at steady-state (1 µM Iso). Eigenvalue decomposition was performed, and the eigenvectors for each timescale were analyzed to identify the most prominent species in each eigenvector (see Materials and Methods). The species of greatest magnitude are shown in Table 2. Certain species deemed to be “fast” using our phase plane method (LCCap, LCCbp, Gsα_gdp_, and Inhib1ptot) were also identified using the Jacobian. However, the tri-modal distribution of timescales (Figure 4) is not observed with the Jacobian approach, because at intermediate timescales each eigenvector is composed of a linear combination of all species. At the same time, some species contribute strongly to several eigenvectors (e.g. B1ARtot). This issue prevents the Jacobian approach from identifying species acting on “medium” timescales (e.g. TnIp, PLBp) that were critical for the 4-variable model reduction. In addition, the Jacobian eigenvectors do not reveal a clear threshold between species found to be “fast” and “medium” by the graphical phase portrait approach.

**Table 2:** Summary of state variable reductions.

*Variable*	*Reduction Process*
(1) L (Iso)	(alg) Converted to parameter with assumption L≈Ltot
(2) β1AR_free_	(alg) Solved analytically using conservation of mass
(3) Gs	(alg) Converted to parameter with assumption Gs≈Gtot
(4) β1ARtot	(ode) Solved analytically using conservation of mass
(5) β1ARd	(ode) Equation not reduced
(6) β1ARp	(ode) Equation not reduced
(7)Gsα_gtp_tot	(ode) Equation not reduced
(8) Gsα_gdp_	(ode) Removed with assumption Gsa_gdp_ <<Gs
(9) Gsβγ	(ode) Removed with assumption Gsβγ ≈ Gsα_gtp_tot
(10) Gsα_gtp_	(alg) Converted to an explicit linear equation based on Gsα_gtp_tot using conc. clamps
(11) Fsk	(alg) Converted to parameter with assumption Fsk≈Fsktot
(12) AC	(alg) Solved analytically using conservation of mass
(13) PDE	(alg) Solved analytically using conservation of mass
(14) IBMX	(alg) Converted to parameter with assumption IBMX≈IBMXtot
(15) cAMPtot	(ode) Equation not reduced
(16) cAMP_free_	(alg) Converted to an explicit power equation based on cAMPtot using conc. clamps
(17) PKA1	(alg) Converted to a Hill equation based on cAMPtot using conc. clamps
(18) PKA2	(alg) Converted to a Hill equation based on cAMPtot using conc. clamps
(19) PLBp	(ode) Reduced to a Hill equation based on PKA1 using conc. clamps (4-var model only)
(20) Inhib1ptot	(ode) Reduced to a Hill equation based on PKA1 using conc. clamps
(21) Inhib1p	(alg) Converted to a Hill equation based on PKA1 using conc. clamps
(22) PP1	(alg) Analytically solved by rearranging parameters
(23) LCCap	(ode) Reduced to a Hill equation based on PKA2 using conc. clamps
(24) LCCbp	(ode) Reduced to a Hill equation based on PKA2 using conc. clamps
(25) TnIp	(ode) Reduced to a Hill equation based on PKA1 using conc. clamps (4-var model only)

## Discussion

Here, we developed a graphical approach to model reduction based on analysis of hysteresis in phase plane trajectories. This approach has the advantage of incorporating the nonlinear dynamics of the system while being graphically intuitive. While timescale decompositions generally produce implicit algebraic equations with numerical difficulties [18], we used “concentration-clamps” to estimate explicit algebraic relationships as part of the model reduction. To test the practical utility of these approaches, we simplified a 25-variable model of β_1_-adrenergic signaling to obtain reasonably accurate 6- or 4-variable reduced order models, even under new perturbations. The 4-variable model was somewhat less accurate than the 6-variable model, consistent with the lesser degree of timescale separation of the last 2 species, TnIp and PLBp. This analysis suggests that overall dynamics of the β-adrenergic signaling pathway can be captured experimentally using a limited number of existing fluorescent reporters focusing on the β-adrenergic receptor [26], G_s_ protein [27], cAMP [28] and certain PKA substrates [29].

Most past work on timescale analysis of biochemical systems has focused on decomposition of the Jacobian matrix. Jacobian-based approaches do have advantages, such as using a single set of calculations for a given steady-state and their scalability to large systems [19]. But the matrix decompositions can be quite complex and vary significantly from one variant of this approach to the next [9], [10], [17], [30], [31]. On the other hand, not all disadvantages previously attributed to dynamic timescale approaches (such as described here) necessarily hold. While dynamical approaches do require simulation and depend on initial conditions [19], resting steady-state initial conditions are quite a reasonable choice, requiring only a single simulation (as performed here). The main advantages of the graphical timescale approach described here are: 1) analysis of the system's nonlinear dynamics rather than a particular linearized steady-state; and 2) intuitive graphical nature, easing both implementation and analysis. Indeed, the graphical timescale decomposition approach does not require a model *per se*; it could be applied directly to high-throughput kinetic data [32], [33].

While Jacobian-based approaches are focused on identifying timescales, the phase-plane approach introduced here instead identifies timescale separation between particular variables. The area of the phase-plane loop is sensitive to both the timescale itself and delays between variables acting on the same timescale. While a limitation for timescale identification, this turns out to be an advantage for model reduction. Indeed, recent Jacobian approaches that have been adapted for dynamic timescale analysis [16], [31] have not shown such substantial model reduction with fully retained network dynamics as shown here.

Kaufmann *et al.* previously used phase plane analysis together with correlation coefficents to examine timescale separation in the red blood cell metabolic network [34]. However, these correlation coefficients miss nonlinear relationships (as were common in the β_1_-adrenergic signaling model), and the metabolite pools were not used to obtain a reduced-order model as shown here. Here, our analysis used transient inputs, generating closed phase-loops enabling quantification of hysteresis between the species of interest. The current graphical model reduction approach should be equally applicable to oscillating systems such as cardiac pacemaking [35] or cell cycle [36], in which case an external input is not required to form a closed phase-loop. Some nonlinear systems exhibiting multistability may not exhibit closed phase-loops; this indicates that algebraic reduction of certain variables may not be appropriate. Indeed, observation of non-closed phase portraits would be a simple way to identify some multi-stabilities.

Model reduction is a central challenge to multiscale modeling in biology [7]. It will be important to integrate the proposed timescale reduction approach with spatial model reduction, such as moment-closure and probability density approaches used for excitation-contraction coupling [37]. Singular perturbation analysis can be applied in space rather than time when certain species diffuse more quickly others [38]. Therefore, it is possible that extensions of the current graphical phase-plane reduction approach may apply to spatial problems as well, where species are plotted against not only other species but also the same species in different subcellular or tissue regions. Finally, complex multiscale models will ultimately need to switch automatically between complex and reduced models at various scales [8], striking a balance between computational requirements and accuracy where appropriate. These advances will undoubtedly be important for multiscale models to reveal fundamental multiscale biological insights and achieve clinical application.

## Materials and Methods

### Timescale Separation Using Phase Portraits

The β-adrenergic model [20], [21] was simulated starting from resting initial conditions and applying a transient 1 µM Iso stimulus for 400 seconds and then 400 additional seconds where Iso = 0 µM. The timecourse for each species was normalized using x_norm_(t) = (x(t)-x_min_)/(x_max_-x_min_), which makes the normalized variable vary between 0 and 1. The purpose of normalization was to standardize the data for measurement of reaction speed, as the species concentrations varied widely in order of magnitude. Concentrations of most species increased during Iso application and decreased after withdrawal. Phase portraits could then be constructed by graphing the various species pairs against each other. In many of the differential equation relationships, one species increases faster than another due to a lag between reaction steps, until the other species catches up at steady-state. With the removal of the ligand, the lag now occurs in the opposite direction; this causes the phase plots for the “drug on” and “drug off” simulations to exhibit hysteresis, with more enclosed area during greater timescale separation. A normalized phase portrait area of 1 corresponds to complete timescale separation, while an area of 0 indicates no timescale separation.

For each pair of variables X and Y, the phase portrait area for the combined “drug on” and “drug off” simulation is computed using the midpoint integration rule as:
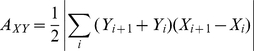
(4)


Note that this simple integration approach does not require uniform spacing of data points in X, Y or time, as long as the shape of the phase loop is accurately characterized. This integration also accounts for situations where the phase loop switches concavity, as occurred for several species that exhibited adaptive responses to isoproterenol (e.g. cAMP_tot_ vs. Gsα_GTP_tot).

### Reducing Differential Equations

Several relationships determined to have a phase portrait area less than the desired timescale separation threshold were simplified using a “concentration clamp” procedure, followed by fitting to an explicit algebraic relationship. Fitting relationships to the original phase portrait alone was often insufficient, because the dynamic range of a given species may be limited for that simulation. Therefore, we performed concentration clamps, where one species was held constant while the other 24 species were run to steady-state. The concentration clamp procedure was repeated for a range of concentrations, each time recording the steady-state value of a species of interest, until the steady-state relationship between the two species was well-characterized. Concentration clamps were compared with the original phase portraits and monitored for conservation of mass to ensure accurate determination of steady-state relationships. Once a suitable concentration clamp curve was obtained, an explicit algebraic relationship between two species was obtained by nonlinear least squares fitting (lsqnonlin in MATLAB). Although the concentration clamp procedure was largely automated, intervention was required to select the appropriate equation for fitting (e.g. linear, Hill, exponential etc.). This could be automated as well by fitting to multiple curves and selecting the fit with the least error.

Table 2 summarizes how each variable in the original 25-variable model was reduced or retained, but additional details are provided here. Three differential equations (LCCap, LCCbp, and Inhib1ptot) and 3 implicit algebraic equations (PKA1, PKA2, Inhib1p) were reduced by fitting to a Hill equation [39]:
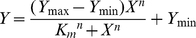
(5)


Two other species (Gsa_gtp_ and cAMPfree) were fitted to concentration clamps using linear and exponential equations, respectively.

Other species were reduced by combining phase portrait areas with additional approximations. For example, β1ARtot rapidly equilibrated with β1ARtot and could be eliminated by conservation of mass. Reassociation of Gsa_gdp_ with Gsβγ was found to be very fast and in the original model, Gsa_gdp_ << Gstot. Therefore this reaction was assumed to be instantaneous. Similarly, Gsβγ was also eliminated, assuming Gsβγ≈Gsα_gtp_tot. Four state variables were removed by assuming that their free concentration was well approximated by their total concentration, which is a parameter. For example, in a typical cell culture experiment the amount of ligand bound to receptor is very small compared with the total amount of ligand, justifying the assumption that [L] ≈ [L]tot. This same assumption was also applied to Gs, IBMX, and forskolin.

Some explicit algebraic equations in the original model needed to be adjusted to account for reductions in other variables. For example, the equation for ligand-receptor complex was changed from 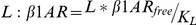 to 
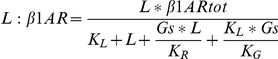
_._

This allowed the equation for β1AR_free_ to be eliminated as it was no longer necessary in the reduced models. Equations and parameters are listed in Text S1.

In the 4-variable reduced model only, two additional differential equations were reduced: TnIp and PLBp. Concentration clamps were used to determine their steady-state relationships with PKA1, but since the relationships were not fast, the steady-state concentration clamp departs somewhat from the corresponding phase portrait (see Figure 5C). These relationships were fit using Hill curves. Equations and parameters are listed in Text S1.

### Timescale Separation by Jacobian Analysis

A similarity transform was applied to decompose the Jacobian into a diagonal matrix Λ, consisting of eigenvalues, and the matrix of eigenvectors M, where 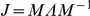
_._ M^−1^ is known as the modal matrix [30]. Each row contains a mode which travels along its respective eigenvalue at the corresponding time scale. A negative eigenvalue represents a relaxing mode [30], [31]. The time scales may be found by taking the inverse of the real part of the eigenvalues: τ_i_ = -1/Real(λ_i_).

The time scales and their respective rows in the modal matrix may be rearranged to order the modes from slowest to fastest (see Table 3). The modes are linear combinations of different variables. The variables of small magnitude were ignored to see which metabolites were responsible for each mode. Modes for timescales ≤1 msec correlated with implicit algebraic relationships in the original model.

**Table 3:** Timescales and eigenvectors determined from the Jacobian matrix.

*Timescales (seconds)*	*Species in Eigenvector*
454.6	B_1_ARd,B_1_ARp
157.7	B_1_ARd,B_1_ARp,B_1_ARtot
46.11	B_1_ARd, B_1_ARp, B_1_ARtot
2.16	cAMPtot, B_1_ARtot, B_1_ARp, B_1_ARd, TnIp, Gsα_gtp_ tot
1.95	B_1_ARtot,Gsα_gtp_tot, PLBp, cAMPtot
1.25	B_1_ARd_free_,Gsα_gtp_tot
0.72	LCCbp
0.66	LCCap
0.036	Inhib1ptot
0.006	Gsa_gdp_ , GsBy
≤0.001	Algebraic Relationships

All eigenvectors in the modal matrix were linear combinations of all state variables. However, the variables with a magnitude greater than 0.09 for each timescale are depicted below. The timescales are the reciprocals of the real parts of the eigenvalues.

## Supporting Information

Figure S1 **Global phase portrait of the β_1_-adrenergic network.** While the global phase portrait is a 25-variable dimension space, 2D slices can be taken that illustrate timescale separation between pairs of state variables. Here, all 210 portraits are shown. Above each portrait, a normalized area of the enclosed loop is calculated that quantifies the degree of timescale separation. The normalized phase portrait area can range from 0 to 1, with 0 indicating no timescale separation.
(EPS)

Table S1 **Parameters in Original Model (from Saucerman et al.** [**20]**, [21****] **).**
(DOC)

Table S2 **Initial conditions for 25-variable model.**
(DOC)

Table S3 **Parameters derived for reduced models.**
(DOC)

Table S4 **Initial conditions for reduced 6-variable and 4-variable models.**
(DOC)

Dataset S1 **MATLAB and CellML code for 25-, 6-, and 4-variable β-adrenergic models.**
(TAR.BZ2)

Text S1 **Equations for the 6- and 4-variable reduced β-adrenergic models.**
(DOC)
