# Supplementary material for: Graphical Approach to Model Reduction for Nonlinear Biochemical Networks
Source: PLoS One. 2011 Aug 25;6(8):e23795. doi: 10.1371/journal.pone.0023795 (PMC3162006; doi:10.1371/journal.pone.0023795)
Supplement: Table S1 — Parameters in Original Model (from Saucerman et al. [20] , [21] ). (DOC) [file pone.0023795.s002.doc]

**Table S1. Parameters in Original Model (from Saucerman et al. [20,21]).**

| *Parameter* | *Value* | *Units* |
| --- | --- | --- |
| Ltot | 1.0 | *μ*M |
| β_1_ARsum | 0.0132 | *μ*M |
| Gstot | 3.83 | *μ*M |
| K_l_ | 0.285 | *μ*M |
| K_r_ | 0.062 | *μ*M |
| K_c_ | 33.0 | *μ*M |
| k_barkp_ | 1.1e-3 | s^-1^ |
| k_barkm_ | 2.2e-3 | s^-1^ |
| k_pkap_ | 3.6e-3 | s^-1^*μ*M^-1^ |
| k_pkam_ | 2.2e-3 | s^-1^ |
| k_gact_ | 16 | s^-1^ |
| k_hyd_ | 0.8 | s^-1^ |
| k_reassoc_ | 1.21e3 | s^-1^*μ*M^-1^ |
| ACtot | 49.7e-3 | *μ*M |
| ATP | 5.0e3 | *μ*M |
| PDEtot | 38.9e-3 | *μ*M |
| IBMXtot | 0.0 | *μ*M |
| Fsktot | 0.0 | *μ*M |
| kac_basal | 0.2 | s^-1^ |
| kac_gsa | 8.5 | s^-1^ |
| kac_fsk | 7.3 | s^-1^ |
| k_pde_ | 5.0 | s^-1^ |
| Km_basal_ | 1.03e3 | *μ*M |
| Km_gsa_ | 315.0 | *μ*M |
| Km_fsk_ | 860.0 | *μ*M |
| Km_pde_ | 1.3 | *μ*M |
| K_gsa_ | 0.4 | *μ*M |
| K_fsk_ | 44.0 | *μ*M |
| Ki_ibmx_ | 30.0 | *μ*M |
| PKA1tot | 0.59 | *μ*M |
| PKA2tot | 0.059 | *μ*M |
| PKItot | 0.18 | *μ*M |
| K_a_ | 9.14 | *μ*M |
| K_b_ | 1.64 | *μ*M |
| K_d_ | 4.375 | *μ*M |
| Ki_pki_ | 0.2e-3 | *μ*M |
| ε_plb_ | 10 | none |
| PLBtot | 106 | *μ*M |
| PP1tot | 0.89 | *μ*M |
| Inhib1tot | 0.3 | *μ*M |
| kpka_plb | 54 | s^-1^ |
| Kmpka_plb | 21 | *μ*M |
| kpp1_plb | 8.5 | s^-1^ |
| Kmpp1_plb | 7.0 | *μ*M |
| kpka_i1 | 60 | s^-1^ |
| Kmpka_i1 | 1.0 | *μ*M |
| Vmaxpp2a_i1 | 14.0 | *μ*M |
| Kmpp2a_i1 | 1.0 | *μ*M |
| Ki_inhib1_ | 1.0e-3 | *μ*M |
| LCCtot | 0.025 | *μ*M |
| PKA2lcctot | 0.025 | *μ*M |
| PP1lcctot | 0.025 | *μ*M |
| PP2Alcctot | 0.025 | *μ*M |
| kpka_lcc | 54 | s^-1^ |
| Kmpka_lcc | 21 | *μ*M |
| kpp1_lcc | 8.52 | s^-1^ |
| Kmpp1_lcc | 3.0 | *μ*M |
| kpp2a_lcc | 10.1 | s^-1^ |
| Kmpp2a_lcc | 3.0 | *μ*M |
| TnItot | 70 | *μ*M |
| PP2Atni | 0.67 | *μ*M |
| kcatpka_tni | 54 | s^-1^ |
| Kmpka_tni | 21 | *μ*M |
| kcatpp2a_tni | 10.1 | s^-1^ |
| Kmpp2a_tni | 4.1 | *μ*M |
