# Supplementary material for: Graphical Approach to Model Reduction for Nonlinear Biochemical Networks
Source: PLoS One. 2011 Aug 25;6(8):e23795. doi: 10.1371/journal.pone.0023795 (PMC3162006; doi:10.1371/journal.pone.0023795)
Supplement: Table S2 — Initial conditions for 25-variable model. (DOC) [file pone.0023795.s003.doc]

**Table S2. Initial conditions for 25-variable model.**

| *Variable* | *Value* | *Units* |
| --- | --- | --- |
| 1. L (Iso) | 1.000E+00 | *μ*M |
| 1. β1AR_free_ | 1.074E-02 | *μ*M |
| 1. Gs | 3.829E+00 | *μ*M |
| 1. β1ARtot | 1.198E-02 | *μ*M |
| 1. β1ARd | 1.223E-12 | *μ*M |
| 1. β1ARp | 1.218E-03 | *μ*M |
| 1. Gsα_gtp_tot | 2.491E-02 | *μ*M |
| 1. Gsα_gdp_ | 6.446E-04 | *μ*M |
| 1. Gsβγ | 2.555E-02 | *μ*M |
| 1. Gsα_gtp_ | 2.229E-02 | *μ*M |
| 1. Fsk | 0.000E+00 | *μ*M |
| 1. AC | 4.708E-02 | *μ*M |
| 1. PDE | 3.890E-02 | *μ*M |
| 1. IBMX | 0.000E+00 | *μ*M |
| 1. cAMPtot | 8.729E-01 | *μ*M |
| 1. cAMP_free_ | 2.258E-01 | *μ*M |
| 1. PKA1 | 6.211E-02 | *μ*M |
| 1. PKA2 | 1.605E-02 | *μ*M |
| 1. PLBp | 4.506E+00 | *μ*M |
| 1. Inhib1ptot | 5.523E-02 | *μ*M |
| 1. Inhib1p | 6.608E-05 | *μ*M |
| 1. PP1 | 8.348E-01 | *μ*M |
| 1. LCCap | 4.316E-03 | *μ*M |
| 1. LCCbp | 4.967E-03 | *μ*M |
| 1. TnIp | 2.492E+00 | *μ*M |
