# Supplementary material for: Graphical Approach to Model Reduction for Nonlinear Biochemical Networks
Source: PLoS One. 2011 Aug 25;6(8):e23795. doi: 10.1371/journal.pone.0023795 (PMC3162006; doi:10.1371/journal.pone.0023795)
Supplement: Table S3 — Parameters derived for reduced models. (DOC) [file pone.0023795.s004.doc]

**Table S3**. Parameters derived for reduced models.

| *Parameter* | *Value* | *Units* |
| --- | --- | --- |
| PKA1max | .8043 | *μ*M |
| PKA1min | -0.0261 | *μ*M |
| n_pka1_ | 1.6368 | none |
| Km_pka1_ | 3.2068 | *μ*M |
| PKA2max | 0.0954 | *μ*M |
| PKA2min | -0.0064 | *μ*M |
| n_pka2_ | 1.8235 | none |
| Km_pka2_ | 1.7547 | *μ*M |
| a_cAMP_ | 0.2715 | none |
| m_cAMP_ | 1.4885 | none |
| k_Gsαgtp_ | 0.9187 | none |
| n_inhib1ptot_ | 1.1113 | none |
| Km_inhib1ptot_ | 0.2335 | *μ*M |
| Inhib1pmax | 5.0717e-4 | *μ*M |
| n_inhib1p_ | 1.1105 | none |
| Km_inhib1p_ | 0.3370 | *μ*M |
| Km_lccap_ | 0.0746 | *μ*M |
| Km_lccbp_ | 0.0630 | *μ*M |
| n_plbp_ | 3.0 | none |
| Km_plbp_ | 0.1555 | *μ*M |
| n_tnip_ | 3.1770 | none |
| Km_tnip_ | 0.1891 | *μ*M |
