# Supplementary material for: Graphical Approach to Model Reduction for Nonlinear Biochemical Networks
Source: PLoS One. 2011 Aug 25;6(8):e23795. doi: 10.1371/journal.pone.0023795 (PMC3162006; doi:10.1371/journal.pone.0023795)
Supplement: Table S4 — Initial conditions for reduced 6-variable and 4-variable models. (DOC) [file pone.0023795.s005.doc]

**Table S4. Initial conditions for reduced 6-variable and 4-variable models.**

| *Variable* | *6-var. Value* | *4-var. Value* | *Units* |
| --- | --- | --- | --- |
| 1. β1ARd | 1.223E-12 | 1.223E-12 | *μ*M |
| 1. β1ARp | 1.218E-03 | 1.218E-03 | *μ*M |
| 1. Gsα_gtp_tot | 2.491E-02 | 2.491E-02 | *μ*M |
| 1. cAMPtot | 8.729E-01 | 8.729E-01 | *μ*M |
| 1. PLBp | 4.506E+00 | *n/a* | *μ*M |
| 1. TnIp | 2.492E+00 | *n/a* | *μ*M |
