# Supplementary material for: Graphical Approach to Model Reduction for Nonlinear Biochemical Networks
Source: PLoS One. 2011 Aug 25;6(8):e23795. doi: 10.1371/journal.pone.0023795 (PMC3162006; doi:10.1371/journal.pone.0023795)
Supplement: Text S1 — Equations for the 6- and 4-variable reduced β-adrenergic models. (DOC) [file pone.0023795.s007.doc]

**Text S1 for:**

**Graphical Approach to Model Reduction for Nonlinear Cell Signaling Networks**

David O. Holland, Nicholas C. Krainak, and Jeffrey J. Saucerman

**Equations for the 6-Variable Model**

$$\beta1ARtot=\beta1ARsum-\beta1ARd-\beta1ARp$$

$$\frac{d\beta1ARd}{dt}=k_{barkp}(LR+LRG)-k_{barkm}\beta1ARd$$

$$LR=\frac{L*\beta1ARtot}{K_{L}+L+\frac{Gs*L}{K_{R}}+\frac{K_{L}*Gs}{K_{G}}}$$

$$LRG=\frac{L*\beta1ARtot}{K_{L}K_{R}+K_{R}L+L*Gs+\frac{K_{L}K_{R}Gs}{K_{G}}}$$

$$RG=\frac{Gs*\beta1ARtot}{K_{G}+Gs+\frac{K_{G}Gs*L}{K_{R}K_{L}}+\frac{K_{G}L}{K_{L}}}$$

$$\beta1ARfree=\beta1ARtot-LR-RG-LRG$$

$$\frac{d\beta1ARp}{dt}=k_{pkap}PKA1*\beta1ARtot-k_{pkam}*\beta1ARp$$

$$\frac{dGsa_{gtp}tot}{dt}=k_{gact}(RG+LRG)-k_{hyd}Gsa_{gtp}tot$$

$$Gs\beta\gamma=Gs\alpha_{gtp}tot$$

$$Gsa_{gtp}=k_{Gsa_{gtp}}Gsa_{gtp}tot$$

$$AC=ACtot-Gsa_{gtp}AC-FskAC$$

$$PDE=PDEtot-\frac{PDEtot*IBMX}{K_{i\_ibmx}+IBMX}$$

$$Gsa_{gtp}AC=\frac{ACtot*Gsa_{gtp}}{K_{gsa}+Gsa_{gtp}+\frac{K_{gsa}Fsk}{K_{fsk}}}$$

$$\begin{matrix} \frac{dcAMPtot}{dt}=\frac{k_{AC,basal}AC*ATP}{Km_{basal}+ATP}+\frac{k_{AC,gsa}Gsa_{gtp}AC*ATP}{Km_{gsa}+ATP}+\frac{k_{AC,fsk}FskAC*ATP}{Km_{fsk}+ATP} \\ -\frac{k_{pde}PDE*cAMPfree}{Km_{pde}+cAMPfree} \end{matrix}$$

$$cAMPfree=a_{cAMP}cAMPtot^{m_{cAMP}}$$

$$PKA1=\frac{(PKA1_{\max}-PKA1_{\min})cAMPtot^{n_{PKA1}}}{Km_{PKA1}^{n_{PKA1}}+cAMPtot^{n_{PKA1}}}+PKA1_{\min}$$

$$PKA2=\frac{(PKA2_{\max}-PKA2_{\min})cAMPtot^{n_{PKA2}}}{Km_{PKA2}^{n_{PKA2}}+cAMPtot^{n_{PKA2}}}+PKA2_{\min}$$

$$\frac{dPLBp}{dt}=\frac{k_{pka,plb}PKA1*PLB}{Km_{pka,plb}+PLB}-\frac{k_{plb,pp1}PP1*PLBp}{Km_{plb,pp1}+PLBp}$$

$$PLB=PLBtot-PLBp$$

$$PP1=\frac{Ki_{inhib1}PP1tot}{Ki_{inhib1}+Inhib1p}$$

$$Inhib1ptot=\frac{Inhib1tot*PKA1^{n_{i1ptot}}}{Km_{i1ptot}^{n_{i1ptot}}+PKA1^{n_{i1ptot}}}$$

$$Inhib1p=\frac{Inhib1p_{\max}PKA1^{n_{i1p}}}{Km_{i1p}^{n_{i1p}}+PKA1^{n_{i1p}}}$$

$$LCCap=\frac{Lcctot*PKA2}{Km_{LCCap}+PKA2}$$

$$LCCbp=\frac{LCCtot*PKA2}{Km_{LCCbp}+PKA2}$$

$$\frac{dTnIp}{dt}=\frac{kcat_{pka,TnI}PKA1*TnI}{Km_{pka,TnI}+TnI}-\frac{kcat_{pp2a,TnI}PP2Atni*TnIp}{Km_{pp2a,TnI}+TnIp}$$

$$TnI=TnItot-TnIp$$

**Altered Equations for the 4-Variable Reduced Model**

$$PLBp=\frac{PLBtot*PKA1^{n_{plbs}}}{Km_{plbs}^{n_{plbs}}+PKA1^{n_{plbs}}}$$

$$TnIp=\frac{TnItot*PKA1^{n_{TnIp}}}{Km_{TnIp}^{n_{TnIp}}+PKA1^{n_{TnIp}}}$$
